# MetaID: A novel method for identification and quantification of metagenomic samples

**DOI:** 10.1186/1471-2164-14-S8-S4

**Published:** 2013-12-09

**Authors:** Satish M Srinivasan, Chittibabu Guda

**Affiliations:** 1 Department of Genetics, Cell Biology and Anatomy, University of Nebraska Medical Center, Omaha, NE 68198-5805, USA; 2 Bioinformatics and Systems Biology Core, University of Nebraska Medical Center, Omaha, NE 68198-5805, USA

**Keywords:** MetaID, Metagenomics, Gut microbiome, Personalized medicine

## Abstract

**Background:**

Advances in next-generation sequencing (NGS) technology has provided us with an opportunity to analyze and evaluate the rich microbial communities present in all natural environments. The shorter reads obtained from the shortgun technology has paved the way for determining the taxonomic profile of a community by simply aligning the reads against the available reference genomes. While several computational methods are available for taxonomic profiling at the genus- and species-level, none of these methods are effective at the strain-level identification due to the increasing difficulty in detecting variation at that level. Here, we present MetaID, an alignment-free *n*-gram based approach that can accurately identify microorganisms at the strain level and estimate the abundance of each organism in a sample, given a metagenomic sequencing dataset.

**Results:**

MetaID is an *n*-gram based method that calculates the profile of unique and common *n*-grams from the dataset of 2,031 prokaryotic genomes and assigns weights to each *n*-gram using a scoring function. This scoring function assigns higher weightage to the *n*-grams that appear in fewer genomes and vice versa; thus, allows for effective use of both unique and common *n*-grams for species identification. Our 10-fold cross-validation results on a simulated dataset show a remarkable accuracy of 99.7% at the strain-level identification of the organisms in gut microbiome. We also demonstrated that our model shows impressive performance even by using only 25% or 50% of the genome sequences for modeling. In addition to identification of the species, our method can also estimate the relative abundance of each species in the simulated metagenomic samples. The generic approach employed in this method can be applied for accurate identification of a wide variety of microbial species (viruses, prokaryotes and eukaryotes) present in any environmental sample.

**Conclusions:**

The proposed scoring function and approach is able to accurately identify and estimate the entire taxa in any metagenomic community. The weights assigned to the common *n*-grams by our scoring function are precisely calibrated to match the reads up to the strain level. Our multipronged validation tests demonstrate that MetaID is sufficiently robust to accurately identify and estimate the abundance of each taxon in any natural environment even when using incomplete or partially sequenced genomes.

## Background

The primary goal of metagenomic studies is to accurately identify and quantify the microbial taxa in a community. Advances in high throughput sequencing techniques or NGS have enabled us to obtain DNA samples from mixed genomes of species that inhabit natural environments. These habitats can range from the microflora living in human gut to those that inhabit soils, ponds and lakes, hot springs, ocean floor, etc. The NGS sequencing technology can yield hundreds of millions of short reads sampled from the DNA in a community, which can be used for profiling the taxonomic and phylogenetic composition of microbial community. Recent metagenomic studies have revealed that the knowledge of the microbial composition in the human gut can help understand the critical role played by these organisms in complex human disorders including Obesity [1-3], Diabetes [4], Inflammatory Bowel Disease (IBD) [2,3], and Symptomatic Atherosclerosis (SA) [5]. Especially, low diversity of microbial community has been associated with Diabetes and IBD and an altered microbial community has been associated with SA [5]. It is evident from literature that no particular taxon is universally present in all the habitats [6]. The diversity of the microbial community in an individual dictates how their biological systems are tuned, which in turn determines their health. Therefore, identification and quantification of the microbial community that inhabits human body can help customize healthcare options to fit to an individual, which is referred to as personalized medicine [7].

Phylogenetic characterization of metagenomic samples has been traditionally done using the well-conserved regions of the 16s rRNA genes [8]. Since each organism can be uniquely characterized based on the 16s rRNA gene, aligning reads against the curated reference database of 16s rRNA will help in profiling the microbial diversity [8]. But this approach is very susceptible to the variability in the copy number of 16s gene and amplification biases that are inherent to the PCR (Polymerase Chain Reaction) [9]. Instead, Liu *et al*. proposed MetaPhyler that can classify metagenomic reads based on 31 universal phylogenetic marker genes which are present only once in most of the genomes and are rarely subjected to horizontal gene transfer [9]. On similar grounds, another method, MetaPhlAn was introduced to identify and estimate the relative abundance of organisms in a community. MetaPhlAn's execution is based on high confidence mapping of the reads against a set of clade-specific marker sequences that are predetermined from the coding sequences of microbial clades [3]. On the other hand, methods such as BLAST and MEGAN [10] have tried to exploit the sequence homology but were inefficient as large portions of the reads fail to have a hit in the database. MEGAN, in particular, suffered to make predictions with shorter read length and could not identify species above genus level [10]. A BLAST based method, CARMA, known for searching Pfam domains and families in the metagenomic reads, promised to yield high accuracies but could make use of only a small fraction (about 6%) of reads for classification [11,12]. Recently, many machine learning based methods have gained popularity. Phymm, in combination with Markov models, identifies individual organisms using the oligonucleotide/oligopeptide (similar to *k*-mers) composition. Again Phymm, in combination with BLAST, PhymmBL, results in better prediction accuracies than each of them performing individually [11]. The Support Vector Machine (SVM) based PhyloPythia also makes use of oligonucleotide frequencies to classify the longer sized reads [11]. MetaCV on the other hand classifies short read sequences by first translating them into six-frame peptides and further decomposing them in to *k*-strings (oligopeptides). These *k*-strings are then weighted and selected for taxonomical classification based on their frequency in the pre-built reference protein database [13].

While several computational methods exist for phylogenetic analysis of metagenomic samples, each method can identify microorganisms at different levels in the taxonomical hierarchy. Machine-learning based methods including Phymm, PhymmBL, and PhyloPythia can work better at Phyla/Class/Family or Genus level [11], but fail at the species/strain level identification. Similarly BLAST-based methods, MEGAN and CARMA, and Phylogeny-based methods, namely 16S rRNA and MetaPhyler fail to discriminate well beyond Genus level [9,10,12]. A recent method, MetaPhlAn can discriminate short reads to the species level [3], but to our knowledge, none of the existing methods can identify metagenomic taxa at the strain level. Here, we propose an alignment-free, *n*-gram based tool, MetaID that can identify the metagenomic taxa at the strain level and also quantify the relative composition of each organism in the sample. This method works solely based on the NGS read information with a remarkable accuracy of over 99% even at the strain-level identification, where the difference between the genomes gets to the minimal; thus, making it difficult to discriminate. The novel scoring function employed by us in this study can effectively utilize the *n*-grams in the dataset to discriminate among the genomes of different strains. While, we have used the simulated metagenomic reads from the human gut microbiome to test the model, our methodology is very generic and hence can be applied for phylogenetic analysis of any metagenomic sample.

## Results and discussion

**Figure 1:**
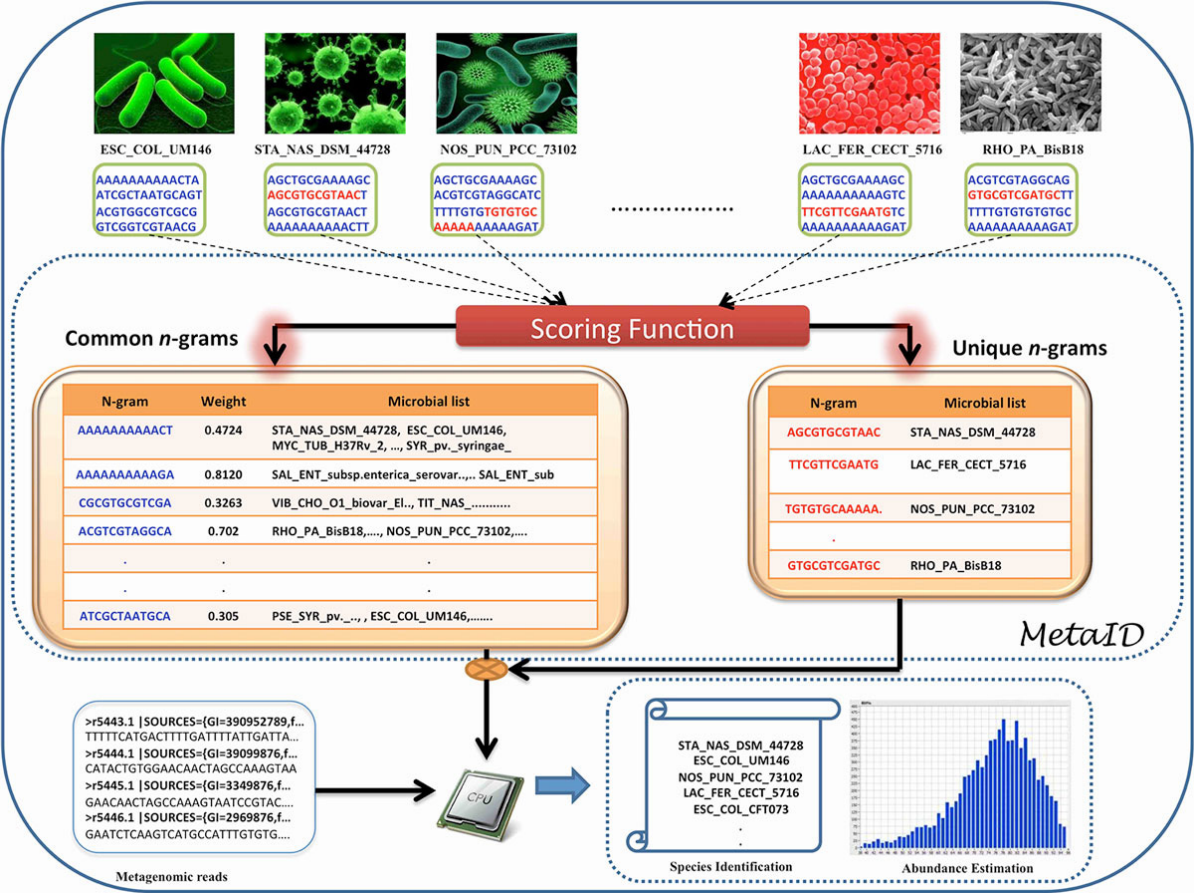
**A schematic diagram showing the methodology and scoring function**. SC_COL_UM146 - *Escherichia coli *UM146, STA_NAS_DSM_44728 - *Stackebrandtia nassauensis *DSM 44728, RHO_PAL_BisB18 - *Rhodopseudomonas palustris *BisB18, LAC_FER_CECT_5716 - *Lactobacillus fermentum *CECT 5716, NOS_PUN_PCC_73102 - *Nostoc punctiforme *PCC 73102

The MetaID method proposed in this study for identifying and quantifying the organisms in the metagenomic reads is based on the *n*-gram model. An *n*-gram is any subsequence of a nucleotide sequence of fixed length *n*. An elaborate description of our scoring function and the *n*-gram model is presented in the Materials and methods section. Figure 1 schematically represents the methodology used in this study. Briefly, the three main steps in the algorithm include model building, identification of the species and quantification of the species. First, a model is built using all the *n*-grams retrieved from all the sequenced microbial genomes. To build the model we used a dataset of 2,031 fully sequenced prokaryotic genomes from the NCBI database.

The *n*-grams from a genome are compared against those from all the other genomes in the dataset to arrive at a comprehensive list of unique (present in only one organism) and common (present in more than one organism) *n*-grams. A scoring function was developed to assign appropriate weights to the unique and common *n*-grams, which can be subsequently used in the testing step. A mixed bag of *n*-grams containing small fractions of the genomes in the dataset was used for testing and optimization of the model. In the second step, *n*-grams obtained from simulated metagenomic sample reads were tested against the model to evaluate the accuracy of the model in identifying the organisms from genus to species to the strain level. Finally, we used a modified abundance estimation method to determine the composition of the metagenomic profile. We also tested the method using the standard performance metrics such as 10-fold cross-validation, using different proportions of genomes to build models and compared the performance of our method against the existing methods, which are described below.

### Determining the optimal value of *n*

**Figure 2:**
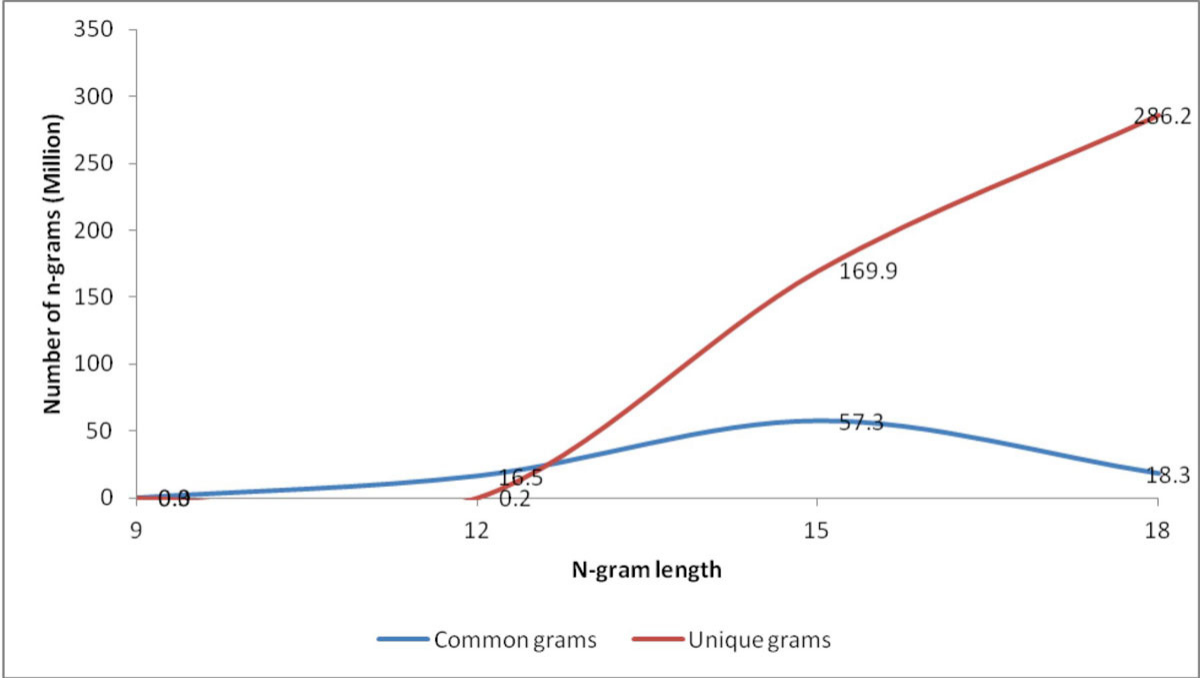
**Number of common and unique *n*-grams as a function of the size of *n***. The sizes of n-grams are varied from 9 to 18 each at a multiple of 3.

In theory, the length of *n*-gram can vary widely, but choosing the optimal length is critical for pragmatic reasons associated with handling of hundreds of millions of NGS reads. Our previous studies [14] demonstrate the limitations with using too small or too large *n*-grams, where smaller *n*-grams loose the discriminative power between the classes, while larger *n*-grams increase the search space exponentially, making it infeasible to build models. To determine an optimal length for the *n*-gram *i.e*. value of *n*, we have tested a randomly chosen subset of 100 genomes in our dataset (Refer to Table S1 in Additional File 1). We retrieved all possible *n*-grams with *n *= 9, 12, 15, and 18 and identified the unique and common *n*-grams across the genomes. The choices for *n *were considered in multiples of 3 because of their biological importance as the coding triplets. Figure 2 shows that at *n *= 9 (common *n*-grams: 0.2 million, unique *n*-grams: 1269) there are fewer numbers of unique *n*-grams that could severely constrain the identification of genomes in the reads. On the other hand, at *n *= 15 (common *n*-grams: 57.3 million, unique *n*-grams: 169.9 million) and *n *=18 (common *n*-grams: 18.3 million, unique *n*-grams: 286.2 million) there was a sudden explosion in the number of unique *n*-grams that could severely challenge the computational capabilities related to model building and testing. But at *n *= 12 (common *n*-grams: 16.4 million, unique *n*-grams: 0.2 million) we obtained a relatively manageable number of common and unique *n*-grams that can help in discriminating between the genomes and at the same time pose no serious threat against model building and testing. Our method requires that a full list of all the *n*-grams and its profile (frequency, weight, etc.) is maintained in the memory (RAM) for fast and efficient processing during the testing and identification phase. Therefore memory storage (RAM size) was a very critical factor for us in determining an appropriate size for the *n*-grams. We used an *n*-gram size of 12 for the rest of this study.

### Model building using 2031 bacterial genomes

Using the *n*-gram size of 12, we identified 16,778,476 common and 140,993 (~141K) unique *n*-grams from 2,031 fully sequenced reference genomes available in the NCBI database as of July 2012. This *n*-gram set represents all possible *n*-grams in the reference genome set that includes a number of distinct strains of the same genus and species. (See Materials and methods section for more information). Since the unique *n*-grams are specific to a genome, they can be used as markers in the identification process, provided such *n*-grams exist for all genomes. Statistical analysis on the 141K unique *n*-grams revealed that, out of 2,031, only 219 genomes contain at least one unique *n*-gram with a range of 26,717 and 1 (Table S2 in Additional File 1). This is not surprising because the number of unique *n*-grams rapidly diminishes from genus to the strain level and our reference genome set contains about 19 organisms that only differ at the strain level. Therefore it is evident that at *n *= 12, we can identify only 219 genomes by using solely the unique *n*-grams resulting in a very low coverage. Instead, we have tapped the common *n*-grams (that exist in more than one reference genome) and employed a novel scoring function to determine the relative weights of each common *n*-gram. This scoring function acts as a dampening factor by assigning higher weightage to the *n*-grams that appear in fewer genomes and vice versa, where the weight rapidly decays for *n*-grams that appear in more number of genomes (Table S3 in Additional File 2). This approach allows for inclusion of all *n*-grams in the model irrespective of their weight, yet differentially weighs the most discriminating *n*-grams from the commonly occurring ones. This attribute of this method makes the model more robust, sensitive and specific in identifying the best fitting reference genome.

**Figure 3:**
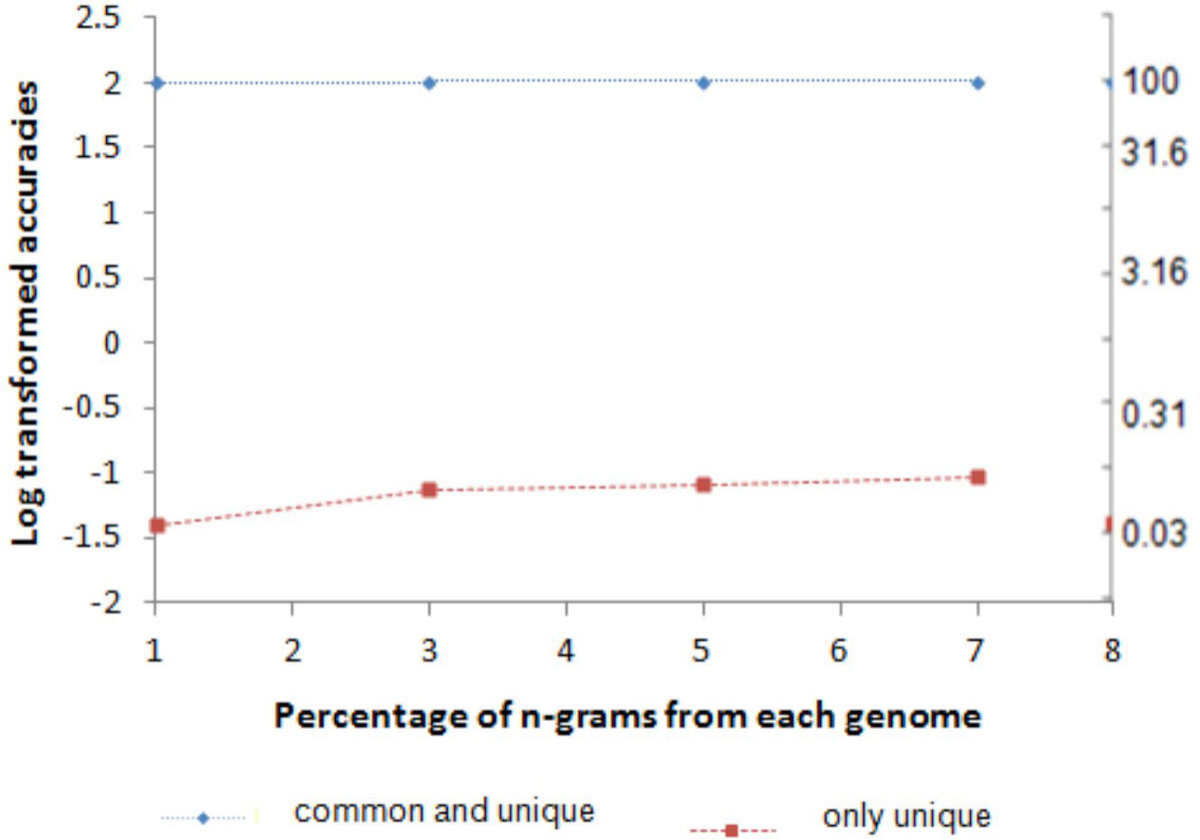
**Comparison of the accuracies across 2,031 bacterial genomes using both the common and unique *n*-grams (*n *= 12) (Model 2) and only unique *n*-grams (*n *= 12) (Model1)**. Scale on the second Y-axis denotes the untransformed accuracies.

The model-building step involves indexing the entire set of common and unique *n*-grams and assigning appropriate weight to each *n*-gram based on its frequency profile across the reference genome set. This model was used for testing the accuracy and optimization of the method. Since the model contains the full set of *n*-grams from the reference genomes, we wanted to test the minimum fraction of *n*-grams needed to accurately identify the organisms at the strain level. From each of the 2,031 genomes, we randomly selected 1%, 3%, 5% and 7% *n*-grams (*n *= 12) and used them to test our model. To demonstrate the power of using weighed common *n*-grams for identification purposes, we tested two different models; (i) using only the unique *n*-grams, and (ii) using both the common and unique *n*-grams. The first model yielded its best accuracy of only 0.09% (Figure 2) when 7% of the *n*-grams were used. In contrast, the second model, with weighted common and unique *n*-grams showed a remarkable and consistent accuracy of 99.23-99.74% (Figure 3), using 1%-7% of the genomic *n*-grams, respectively (Table S4 in Additional File 2). These results suggest two important observations about using the weighted common *n*-grams. First, the accuracy can be improved to the maximum potential and second, only a small fraction of the genomic *n*-grams are required to accurately identify the metagenomic species up to the strain level. In total, using 7% *n*-grams, we found only 5 mispredictions at the strain level. These results strongly demonstrate that the common *n*-grams are vital for accurate identification up to the strain level that is otherwise not possible by using only the unique *n*-grams or a small set of phylogenetic marker genes, which are typically not available beyond the species level. Figure 3 shows the log-transformed accuracies on the y-axis and the corresponding original accuracies on the right hand axis.

Since the bacterial cells harbor a number of plasmid genomes, the presence of similar plasmids across multiple bacterial species/strains may affect the accuracy of our method when applied to predict species from a real metagenomic sample. To test this, we built two separate models, one using only the 2,031 reference genomes without plasmid sequences, and the other, with plasmid sequences (Table S5 in Additional File 2). For testing both models, we used 1% of the total *n*-grams from each bacterial genome, which also contain plasmid sequences as expected in the true metagenomic samples. The prediction accuracy of 99.1% and 98.5% using the model that is built with and without the plasmid sequences respectively shows that our method can aid in better identification of taxa in the NGS metagenomic reads.

### Extending the model to incomplete or partially sequenced genomes

**Figure 4:**
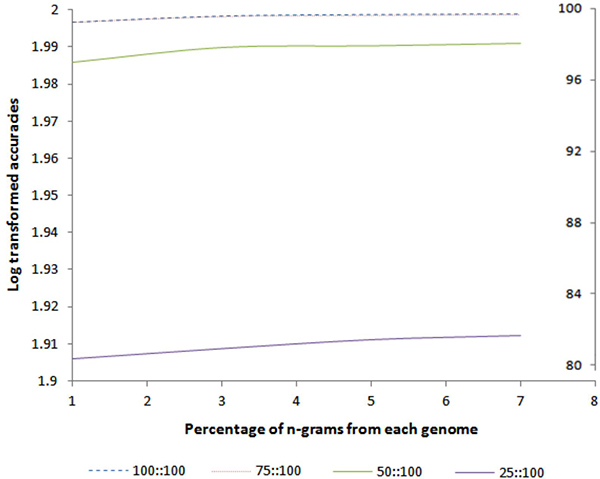
**Accuracies of different models (α::β) using 1, 3, 5 and 7% of the total *n*-grams from each genome**. 75:100 or so forth indicates that the model was built using only 75% of each genome from the reference set and validation was performed using 100% of the genome. Scale on the second Y-axis denotes the untransformed accuracies.

Since a number of genomes are partially sequenced, we are interested to see how this method fares to build models for the identification of partially sequenced genomes. To test this, we performed a (α::β) analysis where, α refers to the % of genome used for model building and β refers to the % of genome used for validation. For example (75:100) indicates that the model was built using only 75% of each genome from the reference set and validation was performed on 100% of the genome. The idea is to train our method with only 75% of the *n*-grams and test it with 100% *n*-grams to see how our method copes around with unseen *n*-grams *i.e*. can our method still identify the genomes with limited knowledge about them. We created partial genomes from our reference genomes set, by randomly selecting α% of the *n*-grams. We built separate models using 100%, 75%, 50% and 25% of the *n*-grams and validated each model using 1%, 3%, 5% and 7% randomly chosen *n*-grams from 100% of the genome. Figure 4 shows the log-transformed accuracies on the y-axis and the corresponding original accuracies on the right hand axis. From Figure 4 it is evident that prediction accuracies are almost identical (above 99%) for models built with 100% and 75% of the genomes at all *n*-gram fractions tested (blue line and the red line overlap with each other). While the models generated using only 50% or 25% of the genomes showed reduced accuracies, the accuracy rates are still very strong *i.e*. 96.8% and 80.5% at 50% and 25% models, respectively, even by testing with only 1% of the total genomic *n*-grams (Table S6 in Additional File 2). Most of the mispredicted taxa using the 25% model were at the level of genus. These results strongly demonstrate that this method can generate accurate models even with partial genomes (up to 50%), and more importantly, only 1% of the genomic *n*-grams are sufficient to identify the species at the strain level with 96.8% accuracy. We attribute the robustness of this method to our scoring function, which uses weighted common *n*-grams to build accurate models for precise identification at the strain level. In contrast, other popular methods such as MetaCV, MEGAN, PhymmBL, NBC, etc., all have reported accuracies below or equal to 89% [13,15] only at the genus level.

### Comparison of performance against other popular methods

We compared the accuracies of our method against other well-known phylogenetic tools. Table 1 presents the genus, species and strain level accuracies of our MetaID against those from NBC, PhymmBL and MEGAN methods. MetaID resulted in a remarkable accuracy of 99.3% compared to NBC, which resulted in only 89% at the strain-level identification. The classification accuracies reported for MetaID and NBC are against a dataset consisting of 2,031 and 635 genomes, respectively. Moving up in the hierarchy, MetaID performed slightly better than NBC across all the levels recording 100% accuracy at the genus level. In comparison to PhymmBL and MEGAN, MetaID's performance was way superior at both the genus and species level. We attribute the superior performance of our method to the underlying *n*-gram model and our scoring function. Since NBC is also based on an *n*-gram model their accuracies at the genus and species level are comparable to ours. The classification accuracies reported for MEGAN and PhymmBL are against a dataset of 737 genomes. It is important here to note that our dataset includes all the genomes that were used to report the accuracies of NBC, MEGAN and PhymmBL.

**Table 1:** Comparison of classification accuracies of NBC, PhymmBL, MEGAN and MetaID

**Serial No**.	Method	Genus-level accuracy	Species-level accuracy	Strain-level accuracy	References
**1**	MetaID	100%	99.95%	99.31%	
**2**	NBC	99.7%	97%	88.8%	[15]
**3**	PhymmBL	85%	76%	NA	[16]
**4**	MEGAN	72.8%	68.1%	NA	[16]

*NA indicates Not Available

We also evaluated the performance of MetaID against other comparable tools such as Blastx 2.2.24, MetaCV, Phymm and RAPSearch2 on a dataset consisting of 154 genera (Table S7 in Additional File 1). Compared to MetaID's 100% accuracy at the genus level, all other tools except Phymm could attain a prediction accuracy of at most 64% (Refer Table 2). For Blastx 2.2.24, MetaCV, Phymm and RAPSearch2, we determined balanced accuracy based on the reported sensitivity and specificity values in the literature [13]. In addition, MetaID also identified 804 species and 1534 strains that are classified within the 154 genera with 99.35% and 98.91% accuracies, respectively (Table S7 in Additional File 1). These results strongly demonstrate the superior accuracy and coverage of MetaID in identifying the taxa in the metagenome at the finest resolution.

**Table 2:** Comparisons of classification accuracies of Blastx, MetaCV, Phymm, RAPSearch2 and MetaID

**Serial No**.	Methods	Sensitivity	Specificity	Genus-level accuracy	Species-level accuracy	Strain-level accuracy	References
**1**	Blastx 2.2.	41%	87%	64%	NA	NA	[13]
**2**	MetaCV	41%	80%	60.5%	NA	NA	[13]
**3**	Phymm	24%	26%	25%	NA	NA	[13]
**4**	RAPSearch2	41%	86%	63.5%	NA	NA	[13]
**5**	MetaID	NA	NA	100%	99.35%	98.91%	

* **NA **indicates Not Available

### Abundance estimation

In addition to identification, MetaID also estimates the relative abundance of different organisms in a given metagenomic sample. To evaluate the performance of our tool, we recruited two mock communities namely mock-even (with equal relative abundance) and mock-staggered (with distributed relative abundance) (Refer to Materials and methods section). We choose mock communities because it is difficult to evaluate results obtained from the real metagenomic samples due to the lack of "golden truth" reference [17]. In the mock-even community, MetaID demonstrated accurate identification of 166 genomes (out of 167) with an accuracy of 99.4%. In addition, MetaID estimated the relative abundance of 166 genomes with 91.5% of the genomes having a relative abundance within 10% deviation from the expected value of 1% (Table S8 in Additional File 1). In contrast, MetaPhlAn could estimate only 75% of the genomes to have a relative abundance within 10% deviation [3]. This result strongly demonstrates that MetaID has a superior performance in comparison to MetaPhlAn for taxonomic profiling.

**Figure 5:**
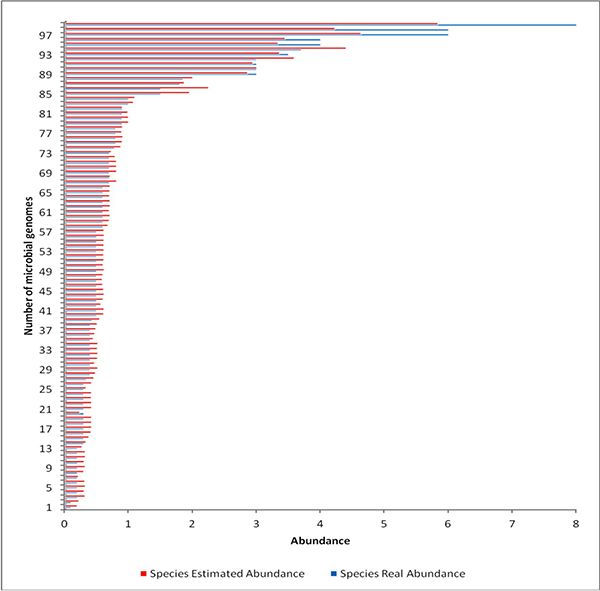
**Comparison of the original and estimated abundances (relative percentage) for 100 microbial genomes in the mock-staggered dataset**.

In contrast to the mock-even communities, the real metagenomic samples contain species with widely varying relative abundances. Therefore, using MetaSim, we simulated a mock-staggered community containing 100 genomes with their original abundance varied between 0.1% and 10%. Using this dataset, MetaID was able to identify all the genomes with 100% accuracy and also estimated the relative abundance of 99 genomes within ± 12% for less abundant genomes *i.e*. between 0.1 to 1.0%, and within ± 3% for highly abundant genomes *i.e*. above 1.0% to 10% (Table S9 in Additional File 1). Figure 5 presents a bar chart comparison of the original and the estimated abundance of the genomes in the mock staggered community. Note that some of the variation in the estimated abundances can be attributed to various factors including the quality of the sequenced genomes, the sensitivity of the error model (substitution, insertion or deletion errors), the quality of the generated reads and the disproportion in the quantity of the reads generated by MetaSim. One peculiar observation is that for *Mycobacterium tuberculosis *H37Ra, with a genome size of 4,419,977, and original abundance of 6.0%, MetaSim generated only 19,925 reads compared to generating 19,556 reads for a similar sized genome *Leptospira interrogans serovar *Lai str. 56601_I with an abundance of 0.5%. Here it is highly possible that either the *Mycobacterium tuberculosis *H37Ra genome is poorly sequenced or there is some inherent computational complexity in the MetaSim.

### Computational complexity of the method

The execution of MetaID involves the following steps: model building using the scoring function, identification of genomes in the metagenomic reads and obtaining their abundance estimates.

Our scoring function performs two major steps. It initially generates the entire set of *n*-grams (*n *= 12) from the nucleotide sequences across different genomes. Secondly, it compares the *n*-grams in a genome against those from all the other genomes in the dataset to arrive at a comprehensive list of unique and common *n*-grams. For generating the *n*-grams from *k *number of genomes with *l *as the length of the longest genome, the worst run-time complexity can be given as O(*knl*). Upon obtaining the *n*-grams, our scoring function compares *n*-grams across genomes using a hashing function. If the largest genome has α number of non-repeatable *n*-grams then the worst time complexity for model building can be given as O(*nk*(*l *+ *α*)). Since model building is a onetime affair and is performed offline, the time complexity of this step does not affect the run-time complexity of our method.

For identifying the genomes in the metagenomic reads our method compares the *n*-grams (*n *= 12) obtained from the reads against the common and unique *n*-grams in the model. For generating the *n*-grams from τ number of reads with *l *as the length of the longest read, the worst run-time complexity can be given as O(τ*nl*). Since comparison of *n*-grams is hash-based, the time complexity for comparing β number of *n*-grams is O(β*n*). Once a matching *n*-gram is found in our model the row entries are updated across all the *δ *number of genomes (columns) either with the weight of the *n*-gram or with a 0 (zero). The worst time-complexity for updating the row and column entries and for obtaining the entire column sum is O(2βδ). At the same time, the worst time-complexity for determining the largest column sum out of all the *δ *columns is O(*δ*). Therefore, the worst time-complexity for this step is O(τ*nl*) +O(*δ*β((*n*+2)+ 1/β)).

For estimating the abundances our method performs an intersection operation on the *n*-grams obtained from the reads against the *n*-grams in the genome. The average time-complexity for the intersection operation on η and λ number of *n*-grams in reads and genome respectively across *k *number of genomes is O(k*min(λ, η)). In the worst case scenario the time-complexity is O(*k*ηλ).

## Conclusions

Here, we have developed an alignment-free *n*-gram based tool, MetaID for determining the taxonomic composition of the microbial community. From the dataset of 2,031 prokaryotic genomes, our method successfully obtained a rich set of common and unique *n*-grams (*n *= 12) and weighted them based upon their natural frequency of occurrence across the genomes. Using these weighted *n*-grams; MetaID was able to demonstrate a classification accuracy of over 99% at the strain level. In comparison to other phylogenetic tools, MetaID was able to classify genomes up to 100% accuracy at the genus level. In addition, MetaID also demonstrated its capability for classifying incomplete or partially sequenced genomes. For estimating the abundances of the genomes in the mock-even community, MetaID demonstrated far superior performance than its variant MetaPhlAn. On the other hand, in the mock-staggered community MetaID demonstrated its ability to estimate less abundant genomes with a deviation of 12% and highly abundant genomes with a 3% deviation from the expected. These results clearly demonstrate that MetaID is capable for taxonomic profiling of metagenomic communities and is generic enough to be applied to a wide variety of microbial species (viruses, prokaryotes and eukaryotes) present in any environmental sample.

## Methods

### Datasets

The input genome dataset consists of a catalogue of 2,031 completely sequenced genomes retrieved from NCBI (ftp://ftp.ncbi.nih.gov/genomes/Bacteria/) in July 2012. The nucleotide sequences from the 2,031 bacterial genomes spans across 292 genera, 537 species and 1,246 strains. After downloading the entire dataset for the bacterial genomes all the plasmid sequences for the respective bacterial genomes were removed. Each of the 2,031 genomes was tagged using the first three letters of their genus and species names. In addition, the entire strain name was retained for clarity purpose. For example, the genome Chlamydia trachomatis D/UW-3/CX was tagged as CHL_TRA_ D/UW-3/CX.

Table S10 in Additional File 1 lists the entire set of 2,031 genomes and their associated statistics such as the length of the genome, the number of *n*-grams (*n *= 12) in the genome, the number of unique and common *n*-grams in the genome and the repeat ratio.

### The *n*-gram model for nucleotide representation

An *n*-gram is any subsequence of a nucleotide sequence of fixed length *n*. In literature, these nucleotide subsequences have been called alternatively as *n*-mers, oligonucleotide, oligopeptide, etc. For the purpose of obtaining common and unique *n*-grams across all the 2,031 bacterial genomes, all possible *n*-grams were extracted from each of the genomes in the dataset. Given a dataset of genome sequences *D*, let *d_i _*be the complete nucleotide sequence for an organism *O_i _*in *D *where $d_{i}=\left(s_{1}s_{2}\ldots s_{k}\right)$, where $s_{i}\in \text{Σ}$ where  Σ represent the set of four nucleotide *A*, *G*, *C *and *T*, then a set of $\left(k-n+1\right)$*n*-grams can be obtained from $d_{i}$ as

$g_{1}=\left(s_{1}\ldots s_{n}\right)$, $g_{2}=\left(s_{2}\ldots s_{n+1}\right),...,g_{k-n+1}=\left(s_{k-n+1}\ldots s_{k}\right)$. Using this *n*-gram model, the following property of *n*-grams can be observed.

• There are countable numbers of *n*-grams across all the genomes that are highly abundant. This phenomenon is related to Zipf's law [15].

Here it is important to note that few of the bacterial genomes contain additional letters namely *N*, *R, Y, W, M, S*, or *K *to account for two ambiguous bases in any given position. For example the letter *N *at any given position indicates unknown base, the letter *R *at any given position indicates either *A *or *G*, the letter *Y *at any given position indicates either *C *or *T *and so on. In addition to that letters *B*, *D*, *H *and *V *represents 3-base ambiguities. Therefore, ∑ = {*A*, *C*, *T*, *G*, *N*, *R*, *Y*, *W*, *M*, *S*, *K*, *B*, *D*, *H*, *V*}.

### Unique and common *n*-gram profile

From the entire 2,031 genomes, all possible non-repeating *n*-grams (*n *= 12) were obtained. The *n*-grams from each genome were compared against the *n*-grams in the other genomes to finally arrive at a set of unique (present in a single genome) and common (present in multiple genomes) *n-*grams. The unique *n*-gram set includes two columns - the *n*-gram and the genome in which it is present. On the other hand the common *n*-gram set includes four columns - the *n*-gram, frequency of its occurrence in the entire dataset, its weight assigned by the scoring function and the genomes in which it is present.

### Scoring function

The scoring function obtains a set of common and unique *n*-grams based on the *n*-gram model discussed above. The scoring function is parameterized with the length of the *n-*gram and the target dataset to begin with. The scoring function reads in the nucleotide sequences of each genome, and generates all possible *n-*grams without any repeats. If a nucleotide sequence is of length , then the total number of *n-*grams is given by $\left(k-n+1\right)$. Once all the *n-*grams are generated, the scoring function compares all the *n-*grams from a genome against those from all the other genomes in the dataset. After successful comparison the scoring function determines a profile of all the common and unique *n*-grams in the dataset. All the unique *n*-grams are assigned a weight of unity, *i.e*. 1, and the common *n*-grams are assigned weights using a dampening factor that accounts for how popular the *n*-gram is with respect to the genomes present in the dataset.

For any *n*-gram  x, the dampening factor is given by the expression ${\text{log}}_{e}\frac{\left|c\right|}{\left|c:x\in c\right|}\;/\;{\text{log}}_{e}\left|c\right|$, where  denotes the total number of genomes in the dataset and $\left|\left\{c:x\in c\right\}\right|$ denotes the total number of genomes in which the *n*-gram  x is present. This factor is similar to the term 'weighting' as discussed in our previous study [14]. The damping factor adjusts the weights of the *n*-grams in such a way that popular *n*-grams receive a low weightage and vice-versa. Table S3 in Additional File 2 shows the weights assigned to few hypothetical *n*-grams based upon their frequency of occurrence in the dataset. If the *n*-gram is present only in a single genome then its weight is unity, *i.e*. 1, and if it is present in all the genomes then its weight is zero, *i.e*. 0.

### Model building

The model-building step involves indexing the entire set of common and unique *n*-grams and assigning appropriate weight to each *n*-gram based on its frequency profile across the reference genome set. For model building our tool considers either the entire set (100%) or a partial (75%, 50% and 25%) set of non-repeatable *n*-grams from each genome. For model building using a partial genome set, non-repeating *n*-grams are randomly selected from the genome. The number of *n*-grams selected from each genome is proportionate to their size.

Model building is a very crucial step in MetaID and it is also a time consuming process. In case of adding new genomes to the dataset or adding a completely different community including viral, fungus, archaeal, etc., the model-building step has to be carried out again. Therefore, this update process can be scheduled at periodic intervals. Moreover, model-building step in our tool is an offline process.

### Repeat ratio

While harvesting the *n*-grams (*n *= 12) from the reference genomes we observed that there are a large number of *n*-grams that have the tendency to re-appear. Therefore, we came up with a parameter "repeat ratio" to account for the abundances of repeated *n*-grams in each genome. Repeat ratio is determined by computing the fraction of the repeated *n*-grams to the total number of *n*-grams in the genome. Here repeat ratios are represented as percentages. The Table S11 and Figure S1 (in Additional File 2) presents a histogram of the repeat ratio distribution across the 2,031 bacterial genomes. Across 2,031 bacterial genomes the repeat ratio distribution ranged widely between 0.85% to 71.53%. Only small fractions of the genome, *i.e.*, 3.3% have repeat ratios within 10%. Almost about 69.2% of the genomes have a repeat ratio between 25% and 60%. In total nearly 99.6% of the genomes have their repeat ratios ranging from 10% to 70%. The mean and the standard deviation of the repeat ratios across 2,031 bacterial genomes were observed to be 27.57 and 12.52 respectively.

### Testing and identification (classification)

Though the objectives behind our testing and identification (classification) steps are the same, there is a subtle difference between them. For testing we consider 1%, 3%, 5%, and 7% of the non-repeated *n*-grams randomly chosen from each genome and try to identify their origin. In contrast, for identification we consider the entire set of metagenomic reads to harvest all possible *n*-grams (*n *= 12) and try to determine the constituent organisms in a given community.

Let us consider $R=\left\{g_{1},g_{2},g_{3}\ldots ,g_{n}\right\}$ as a set of *n*-grams obtained from the reads or randomly selected from the genome and $G=\left\{G_{1},G_{2},G_{3}\ldots ,G_{m}\right\}$ as the set of genomes present in the database. We define a mapping from *R *to *G *as R→G where all the elements in domain *R *maps to a single element in co-domain *G i.e*. $g_{1},g_{2},g_{3}\ldots ,g_{n}\to G_{x}$ where $G_{x}$ is the only single range in co-domain  G and $G_{x}\in G$. To obtain a mapping from *R *to *G *we construct a n*m matrix of the form $\left(\begin{array}{ccc} y_{0,0} & \cdots \; & y_{0,m}\\ \vdots & \ddots & \vdots \\ y_{n,0} & \cdots \; & y_{n,m} \end{array}\right)$ where we define $T=\left\{c_{1},c_{2},c_{3}\ldots ,c_{m}\right\}$ where $c_{0}=\left\{y_{0,0},y_{1,0},y_{2,0}\ldots ,y_{n,0}\right\}$, $c_{1}=\left\{y_{0,1},y_{1,1},y_{2,1}\ldots ,y_{n,1}\right\}$, $c_{m}=\left\{y_{0,m},y_{1,m},y_{2,m}\ldots ,y_{n,m}\right\}$ are the columns in the n*m matrix and $y_{e,f}$ represent the weight assigned to an *n*-gram *e *that is present in genome *f *or 0 if the *n*-gram *e *is not present in the genome *f*. In the above-mentioned n*m matrix we define $\sum c_{z}=\sum \left(y_{0,z}+y_{1,z}+y_{2,z}+\ldots +y_{n,z}\right)$ as the sum of all the elements in the column *z*. After computing the sum of each column in the n*m matrix we arrange all the column sums in a descending order. We then associate $g_{1},g_{2},g_{3}\ldots ,g_{n}\to G_{x}$ provided that $\sum c_{x}>\sum c_{x-1}>\sum c_{x-2}>\ldots >\sum c_{1}$ and $\sum c_{x}>\sum c_{x+1}>\sum c_{x+2}>\ldots >\sum c_{m}$.

In summary, after obtaining the *n*-grams from the reads or from the genomes we construct a matrix with the rows representing the *n*-grams and the columns representing the entire set of genomes in the dataset. We then replace each matrix entry with the weight of the *n*-gram corresponding to that particular genome. If an *n*-gram is not part of the genome then we replace that entry with a zero *i.e.*, 0. After filling the matrix entries, we determine the column sum against each genome; identify the highest column sum and associate (map) the entire set of *n*-grams to that particular genome.

It is important to note that in the identification step we try to map a set of reads to a genome instead of mapping each single read to a genome. This is because it is hard to classify each single read to a genome due to the intense computation involvement and lack of discriminatory signals in them. Again, in order to ensure a successful classification we compared our classification results against the classifications performed by MetaSim.

### MetaSim reads

Metagenomic reads for our mock-staggered communities were obtained using the MetaSim simulation tool. On parameterizing MetaSim with the genomes, their abundance profile, the empirical error model (Table S12 in Additional File 2) and the total number of reads to be generated; MetaSim generates a set of reads against each genome. For our mock-staggered community, MetaSim generated about 3 million 100 bp pair-end reads. Table S13 in Additional File 2 shows the parameter settings used in MetaSim for constructing the mock staggered community and the details of the simulation output.

### Mock communities

Two different mock communities were used in this study. The first one is the mock-even community that is constructed from two datasets namely HC1 and HC2 obtained from MetaPhlAn website (http://www.huttenhower.org/metaphlan). The original datasets consisted of 100 genomes each with an equal abundance of 1%. From these datasets, we constructed a mock-even community of 167 microbial genomes (72 from HC1 + 95 from HC2) that are also present in our 2,031 set of reference genomes. The entire set of reads pertaining to these 167 genomes was included in our community to ensure that their abundances are equal *i.e*. 1%. We eliminated the rest 33 genomes either due to their absence in our dataset or because there was no appropriate mapping found between the KEGG ID's in HC1 and HC2 to our NCBI names in the database.

Secondly, we constructed a mock-staggered community by randomly choosing 100 microbial genomes out of the 2,031 genomes in our dataset. The final mock-staggered community included genomes with genome sizes varying between 641,770 to 9,033,684 and with their repeat ratios ranging from 7 to 63. For this community, we randomly assigned an abundance value for each genome between 0.1% and 10% totaling up to 100% (Table S9 in Additional File 1).

### Abundance estimation

Considering a set of reads from a genome, we harvested all possible non-repeated *n*-grams (*n *= 12) and mapped them against their reference genome. Upon mapping, we counted the total number of *n*-grams that is in common (intersection) between the reads and the reference genome. We determined the relative "Observed Abundance" for a genome as the ratio of its number of non-repeated *n*-gram counts to the total sum of the non-repeating *n*-grams of the genomes present in the community multiplied by the total number of genomes in the sample. After determining the observed abundances we noticed that genomes with extreme repeat ratios *i.e*. above 50 or below 15 had a tendency to be estimated higher or lower respectively. Therefore to correct the observed abundances we either subtract or add the first standard deviation of the repeat ratios of 2031 genomes to the mean of the repeat ratios of 2031 genomes. On the other hand, if the repeat ratio of a genome lies between 15 and 50 then the mean of the repeat ratios of 2031 genomes is used as such. The corrected abundance for a genome is reported based on their repeat ratio using the following expressions:

$$Corrected\;\;Abundance=Observed\;\;Abundance-{\text{log}}_{10}\left(\frac{Repeat\;\;Ratio}{\mu_{Repeat\;\;ratio\;\;\;of\;\;2031\;\;genomes}}\right).$$

15<Repeatratio<50.

$Corrected\;\;Abundance=Observed\;\;Abundance-{\text{log}}_{10}\left(\frac{Repeat\;\;Ratio}{\mu_{Repeat\;\;ratio\;\;of\;\;2031\;\;genomes}\pm \sigma_{Repeat\;\;ratio\;\;of\;\;2031\;\;genomes}}\right).$Repeatratio>50andRepeatratio<15.

Where  μ is the mean and  σ is the standard deviation of the repeat ratios for the 2,031 genomes present in our dataset (Table S10 in Additional File 1). Note here that the mean and standard deviation for the repeat ratios will change with the addition or elimination of genomes in the dataset.

From Figure S1 (Additional File 2), we noticed that most of the genomes have their repeat ratio ranging between 15% and 50%. Therefore when correcting the abundances (Corrected Abundance) we subtract one standard deviation from the mean for those genomes whose repeat ratio is above 50% and add one standard deviation to the mean for those genomes whose repeat ratio is below 15%. For the genomes with repeat ratios between 15% and 50%, the mean of the repeat ratio is considered as such. Here we report the abundance estimates for any given community in percentages *i.e*. 100% for the entire community or equal to the number of microbial species in the community. Therefore, if the corrected abundance does not add up to 100% or equal to the number of species we report the "Estimated Abundance" which is normalized to either 100% or equal to the number of species in the community.

### Performance metrics

We report standard performance measure in terms of accuracy as percentages. Accuracy is defined as the ratio of number of entries (genomes) that have been correctly identified to the number of entries under consideration. In some cases, we have reported balanced accuracies wherever we have information about specificity and sensitivity *i.e.*,

$$\text{Balanced}\;\;\text{Accuracy}=\text{½}*\left(\text{sensitivity}+\text{specificity}\right).$$

## List of abbreviations

BLAST: Basic Local Alignment Search Tool; DNA: Deoxyribonucleic acid; FASTA: fast A; HC1: High Complexity evenly distributed metagenome; HC2: High Complexity evenly distributed metagenome; IBD: Inflammatory Bowel Disease; KEGG: Kyoto Encyclopedia of Genes and Genomes; MetaCV: Composition based classification for short metagenomic sequences; MEGAN: Metagenome Analyzer; MetaID: Metagenomic Identification and abundance estimation tool; MetaSim: Sequencing Simulator for Genomics and Metagenomics; NBC: Naïve Bayes Classifier; NGS: Next Generation Sequencing; PCR: Polymerase Chain Reaction; PhymmBL: Hybrid classifier of short metagenomic reads using Interpolated Markov Models and BLAST; RAM: Random-access memory; RAPSearch2: Reduced Alphabet based Protein similarity Search; rRNA: Ribosomal ribonucleic Acid; SA: Symptomatic Atherosclerosis; SVM: Support Vector Machine.

## Competing interests

The authors declare that they have no competing interests.

## Authors' contributions

SMS developed the metagenomic tool MetaID, carried out the analyses and drafted the manuscript. CG conceived the original study, conceptually provided the framework for the MetaID tool and for the project and assisted in the manuscript preparation. Both the authors have read and approved the final manuscript.

## Authors' information

SMS is a Postdoctoral Research Associate with a strong background in computer science. CG (Associate professor) has an interdisciplinary background in molecular and computational biology. He has published a number of computational methods with a variety of applications in biomedical research, since 2001.

## Supplementary Material

Additional File 1 Table S1:
**This file lists the 100 bacterial genomes randomly selected for determining an appropriate size for the *n*-gram**. There are two columns in this file. The two columns are as follows: (1) Full name of the bacterial genome, (2) Code (Genus_species_strain) for respective bacterial genome.
**Table S2:** Number of bacterial species with unique *n*-grams *vs *number of bacterial species in the dataset. Listed below are 219 bacterial genomes containing unique *n*-grams.
**Table S7:** This file lists the 154 bacterial genera selected for the performance evaluation of accuracies across the following tools: MetaID, Blast, MetaCV, Phymm and RAPSearch2. There are three columns in this file. The three columns are as follows: (1) Bacterial genera's, (2) Number of strains in each bacterial genera, (3) Number of species in each bacterial genera.
**Table S8:** This file reports the original and the estimated abundances of 166 (71 in HC1 and 95 in HC2) bacterial genomes in the mock even communities. There are seven columns in this file. The seven columns are as follows: (1) Full name of the bacterial genome, (2) Code (Genus_species_strain) for respective bacterial genome, (3) The KEGG Id's for the bacterial genome, (4) The original abundance of these genomes in the mock even dataset, (5) The number of reads for each genomes, (6) The corrected abundance of the genomes in the mock even dataset, (7) The estimated abundance of the genomes in the mock even dataset.
**Table S9:** This file reports both the original and the estimated abundances of 100 bacterial genomes in the mock staggered community. There are seven columns in this file. The seven columns are as follows: (1) Full name of the bacterial genome, (2) Code (Genus_species_strain) for respective bacterial genome, (3) The original abundance of these genomes in the mock staggered dataset, (4) The number of reads for each genomes generated using the MetaSim, (5) The Observed abundance of the genomes in the mock staggered dataset, (6) The corrected abundance of the genomes in the mock staggered dataset, (7) The estimated abundance of the genomes in the mock staggered dataset.
**Table S10:** This file lists the entire 2031 bacterial genomes present in our database obtained from NCBI. There are totally eight columns in this file. The eight columns are as follows: (1) Full name of the bacterial genome, (2) Code (Genus_species_strain) for respective bacterial genome, (3) Length of the circular genome, (4) Number of *n*-grams in the genome, (5) Number of unique *n*-grams in the genome, (6) Number of common *n*-grams in the bacterial genome, (7) Number of repeated *n*-grams in the genome, (8) Repeat ratio. Click here for file

Additional File 2 Table S3:
**Weights assigned to a hypothetical *n*-gram based upon its frequency in the dataset**.
**Table S4:** Comparison of the accuracies across 2031 bacterial genomes using both the common and unique *n*-grams and only the unique *n*-grams.
**Table S5:** Plasmid sequence testing across 2031 bacterial genomes using 100% genomes.
**Table S6:** Validation accuracies of different Models (α::β) using 1, 3, 5, and 7% *n*-grams from 2031 bacterial genomes.
**Table S11:** Histogram statistics for the repeat ratio distribution across 2031 bacterial genomes.
**Table S12**: Empirical error models and error rate per base.
**Table S13:**: MetaSim parameter settings and simulation details.
**Figure S1:** Histogram of the repeat ratio distribution across 2031 bacterial genome. Click here for file

## Declarations

The publication costs for this article were funded by the corresponding author using the support from NIH.

This article has been published as part of *BMC Genomics *Volume 14 Supplement 8, 2013: Selected articles from the International Conference on Intelligent Biology and Medicine (ICIBM 2013): Genomics. The full contents of the supplement are available online at http://www.biomedcentral.com/bmcgenomics/supplements/14/S8.
