# Supplementary material for: MetaID: A novel method for identification and quantification of metagenomic samples
Source: BMC Genomics. 2013 Dec 9;14(Suppl 8):S4. doi: 10.1186/1471-2164-14-S8-S4 (PMC4042266; doi:10.1186/1471-2164-14-S8-S4)
Supplement: Additional File 2 — Table S3: Weights assigned to a hypothetical n-gram based upon its frequency in the dataset. Table S4: Comparison of the accuracies across 2031 bacterial genomes using both the common and unique n-grams and only the unique n-grams. Table S5: Plasmid sequence testing across 2031 bacterial genomes using 100% genomes. Table S6: Validation accuracies of different Models (α::β) using 1, 3, 5, and 7% n-grams from 2031 bacterial genomes. Table S11: Histogram statistics for the repeat ratio distribution across 2031 bacterial genomes. Table S12: Empirical error models and error rate per base. Table S13:: MetaSim parameter settings and simulation details. Figure S1: Histogram of the repeat ratio distribution across 2031 bacterial genome. [file 1471-2164-14-S8-S4-S2.docx]

**Additional File 2:**

Srinivasan and Guda, 2013

**Table S3**: Weights assigned to a hypothetical *n*-gram based upon its frequency in the dataset

| Number of genomes in which the *n*-gram is present | Weights for the n-gram |
| --- | --- |
| 1 | 1 |
| 2 | 0.908991 |
| 5 | 0.788685 |
| 10 | 0.697676 |
| 50 | 0.486361 |
| 100 | 0.395352 |
| 500 | 0.184037 |
| 1000 | 0.093028 |
| 1500 | 0.039791 |
| 2000 | 0.00202 |
| 2031 | 0 |

**Table S4**: Comparison of the accuracies across 2031 bacterial genomes using both the common and unique *n*-grams and only the unique *n*-grams

| % of n-grams | Using unique and weighted common grams | using unique n-grams only |
| --- | --- | --- |
| 0 | 0 | 0 |
| 1 | 99.23 | 0.0384 |
| 3 | 99.61 | 0.0738 |
| 5 | 99.7 | 0.08124 |
| 7 | 99.74 | 0.09354 |

**Table S5**: Plasmid sequence testing across 2031 bacterial genomes using 100% genomes

| % of n-grams | A | B | C | D |
| --- | --- | --- | --- | --- |
| 1 | 99.23 | 98.52 | 98.96 | 99.13 |
| 3 | 99.61 | 99.01 | 99.45 | 99.55 |
| 5 | 99.7 | 99.06 | 99.65 | 99.65 |
| 7 | 99.74 | 99.06 | 99.7 | 99.7 |

A: Model building and testing of genomes without plasmid sequences

B: Model building without plasmid sequences and testing of genomes with plasmid sequences

C: Model building with plasmid sequences and testing of genomes after removing the plasmid sequences

D: Model building and testing of genomes with plasmid sequences

Analysis are based on 100::100 (100% n-grams used for both model building and testing)

**Table S6**: validation accuracies of different Models (α::β) using 1, 3, 5 and 7% *n*-grams from 2031 bacterial genomes

| % of n-grams | 100::100 | 75::100 | 50::100 | 25::100 |
| --- | --- | --- | --- | --- |
| 1 | 99.23 | 99.21 | 96.79 | 80.55 |
| 3 | 99.61 | 99.6 | 97.68 | 81.04 |
| 5 | 99.7 | 99.68 | 97.78 | 81.48 |
| 7 | 99.74 | 99.71 | 97.93 | 81.68 |

**Table S11**: Histogram statistics for the repeat ratio distribution across 2031 bacterial genomes

| *Bin* | *Frequency* | *Cumulative %* | *Bin* | *Frequency* | *Cumulative %* |
| --- | --- | --- | --- | --- | --- |
| 0 | 0 | 0.00% | 25 | 452 | 22.26% |
| 5 | 4 | 0.20% | 20 | 393 | 41.61% |
| 10 | 65 | 3.40% | 30 | 375 | 60.07% |
| 15 | 116 | 9.11% | 35 | 170 | 68.44% |
| 20 | 393 | 28.46% | 40 | 128 | 74.74% |
| 25 | 452 | 50.71% | 15 | 116 | 80.45% |
| 30 | 375 | 69.18% | 50 | 105 | 85.62% |
| 35 | 170 | 77.55% | 45 | 89 | 90.00% |
| 40 | 128 | 83.85% | 55 | 69 | 93.40% |
| 45 | 89 | 88.23% | 10 | 65 | 96.60% |
| 50 | 105 | 93.40% | 65 | 22 | 97.69% |
| 55 | 69 | 96.80% | 70 | 22 | 98.77% |
| 60 | 18 | 97.69% | 60 | 18 | 99.66% |
| 65 | 22 | 98.77% | 5 | 4 | 99.85% |
| 70 | 22 | 99.85% | 75 | 3 | 100.00% |
| 75 | 3 | 100.00% | 0 | 0 | 100.00% |
| More | 0 | 100.00% | More | 0 | 100.00% |

**Table S12**: Empirical error model and error rate per base

| # <error type>  # Set all substitution rates  SUBSTITUTION_ERROR  0.001 first base  0.001 second base  0.001 .  0.001 .  0.001 .  0.001  0.001  0.001  0.001  0.001  0.0001  0.0001  0.0001  0.0001  0.0001  0.0001  0.0001  0.0001  0.0001  0.0001  0.0001  0.0001  0.0001  0.0001  0.0001  0.0001  0.0001  0.0001  0.0001  0.0001  0.0001  0.0001  0.0001  0.0001  0.0001  0.0001  0.0001  0.0001  0.0001  0.0001  0.0001  0.0001  0.0001  0.0001  0.0001  0.0001  0.0001  0.0001  0.0001  0.0001  0.0001  0.0001  0.0001  0.0001  0.0001  0.0001  0.0001  0.0001  0.0001  0.0001  0.0001  0.0001  0.0001  0.0001  0.0001  0.0001  0.0001  0.0001  0.0001  0.0001  0.0001  0.0001  0.0001  0.0001  0.0001  0.0001  0.0001  0.0001  0.0001  0.0001  0.0001  0.0001  0.0001  0.0001  0.0001  0.0001  0.0001  0.0001  0.0001  0.0001  0.01  0.02  0.05  0.03  0.01  0.01  0.02  0.04 .  0.04 .  0.05 last base |
| --- |

**Table S13**: MetaSim parameter setting and simulation details

| Simulating files:  [profile.mprf]  Simulator Settings:  Preset Name: EMP-1  Number Of Reads / Mate Pairs=3000000  Error Model=Empirical  Empirical Error Model File=/storage_m/ssrinivasan/Metasim_simulation/simulation_2_feb/error.mconf  Read Length=100  Configured Error Functions:  SUBSTITUTION_ERROR  Finger print: c18feb0f6fc3467e8ace46658f5695c4e8d9fa86  Paired end probability=0.0  Read #1 ends at insert end=true  Read #2 ends at insert end=true  Empirical Error Model File 2=/storage_m/ssrinivasan/Metasim_simulation/simulation_2_feb/error.mconf  Read Length=100  Configured Error Functions:  SUBSTITUTION_ERROR  Finger print: c18feb0f6fc3467e8ace46658f5695c4e8d9fa86  Empirical Error Model DNA Clone Parameters=  Distribution: Normal  Mean: 2000.0  2nd parameter: 200.0  Combine All Files=false  Uniform Sequence Weights=false  Number Of Threads=1  Write FastA=true  Compress Output Files=true  Empirical Error Model Run Statistics  Positional Substitution Counts: [3065, 2967, 2968, 2981, 2949, 2986, 2971, 2968, 2990, 3025, 279, 318, 268, 310, 302, 339, 305, 300, 277, 259, 260, 280, 296, 238, 282, 281, 325, 339, 318, 287, 294, 321, 281, 298, 286, 307];  A(A,C): 17054  A(A,G): 17164  A(A,T): 17157  A(C,A): 14535  A(C,G): 14676  A(C,T): 15013  A(G,A): 15835  A(G,C): 15863  A(G,T): 15860  A(T,A): 15999  A(T,C): 16069  A(T,G): 16100  C(A,C): 17151  C(A,G): 17018  C(A,T): 17304  C(C,A): 23503  C(C,G): 23210  C(C,T): 23466  C(G,A): 29150  C(G,C): 29126  C(G,T): 28889  C(T,A): 15762  C(T,C): 15872  C(T,G): 15686  G(A,C): 18481  G(A,G): 18660  G(A,T): 18681  G(C,A): 28509  G(C,G): 28320  G(C,T): 28604  G(G,A): 23323  G(G,C): 23028  G(G,T): 23216  G(T,A): 14918  G(T,C): 14866  G(T,G): 14612  T(A,C): 10996  T(A,G): 10815  T(A,T): 10657  T(C,A): 18683  T(C,G): 18682  T(C,T): 18535  T(G,A): 17149  T(G,C): 17164  T(G,T): 17043  T(T,A): 16862  T(T,C): 16712  T(T,G): 17016  Base Counts:  STRONG: 1  PURINE: 4  ADENINE: 64119645  KETO: 3  THYMINE: 64121362  CYTOSINE: 85885304  AMINO: 1  GUANINE: 85871670  PYRIMIDINE: 5  WEAK: 2  ANY: 2003  Generated 3000000 Reads  Average Read Length is 100.00 Base Pairs  Processed 300000000 Base Pairs  Generated 300000000 Base Pairs  Insertions: 0  Deletions: 0  Substitutions: 892994  Read data saved as `profile-Empirical.17ce3493.fna.gz'.  Done. |
| --- |

**Figure S1**: Histogram of the repeat ratio distribution across 2031 bacterial genome
